# 1780. COVID-19 Vaccine Accessibility and Uptake among U.S. Children Aged 6 Months-4 Years and 5 Years-11 Years by Domains of Social Vulnerability

**DOI:** 10.1093/ofid/ofad500.1609

**Published:** 2023-11-27

**Authors:** Rohan Khazanchi, Benjamin Rader, Jonathan Cantor, Dena M Bravata, Kathleen A McManus, Christopher Whaley, Rebecca Weintraub, John Brownstein

**Affiliations:** Brigham and Women’s Hospital, Boston Children’s Hospital, and Boston Medical Center, Jamaica Plain, Massachusetts; Boston Children's Hospital, Boston, Massachusetts; RAND Corporation, Santa Monica, California; Castlight Health, San Francisco, California; University of Virginia, Charlottesville, Virginia; RAND Corporation, Santa Monica, California; Ariadne Labs, Boston, Massachusetts; Boston Children's Hospital, Boston, Massachusetts

## Abstract

**Background:**

The COVID-19 pandemic, and pandemic-related interventions, have had markedly disparate effects on marginalized populations. We examined whether the inequitable distribution of other COVID-19 resources was similarly reflected within the pediatric vaccination rollout.

**Methods:**

We analyzed from a comprehensive national database of U.S. pediatric vaccination distribution sites and administered doses (VaccineFinder) as of 7/29/2022. We ascertained accessibility by geocoding sites, measuring one-way travel times from every Census tract population center to the nearest site, and weighting tracts by population demographics (rurality, age, race, ethnicity) to obtain nationwide estimates. We used population-weighted quasipoisson regressions adjusted for state fixed effects to compare vaccination uptake between the most and least socially vulnerable quartiles of counties by Social Vulnerability Index (SVI) domains (socioeconomic status, SES; household composition & disability, HCD; minority status & language, MSL; housing type & transportation, HTT).

**Results:**

We identified 15,233,956 total vaccine doses administered across 27,526 sites. Rural, non-Hispanic, White, and Native populations had longer one-way travel times to the nearest pediatric vaccination site than urban, Hispanic, Black, and Asian populations. Greater social vulnerability by overall SVI, SES, and HCD was associated with decreased vaccine uptake among children in the 6mo-4y (Overall: IRR 0.70 [95%CI 0.60-0.81], SES: 0.66 [0.58-0.75], HCD: 0.38 [0.33-0.44]) and 5y-11y (Overall: 0.85 [0.77-0.95], SES: 0.71 [0.65-0.78], HCD: 0.67 [0.61-0.74]) groups, whereas greater social vulnerability by MSL was associated with increased uptake in both age groups (6mo-4y: 5.16 [3.59-7.42], 5y-11y: 1.73 [1.44-2.08]).

**Figure 1.**
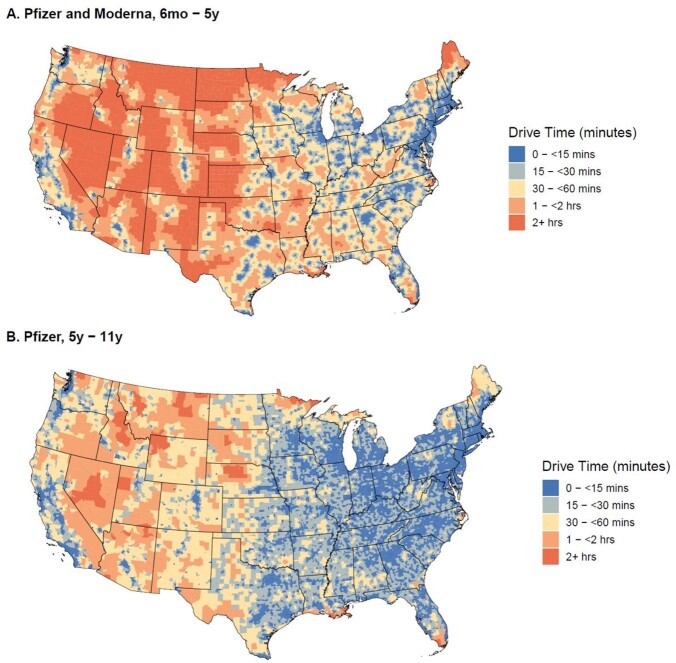
Geospatial Visualization of One-Way Travel Time to the Nearest COVID-19 Vaccination Site by Age

**Figure 2.**
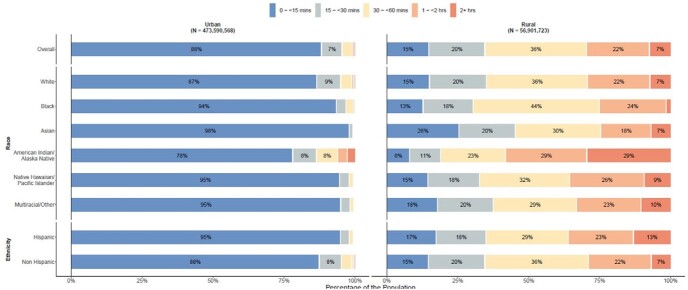
One-Way Travel Time to the Nearest 6mo-4y COVID-19 Vaccination Site by Race, Ethnicity, Rurality

**Figure 3.**
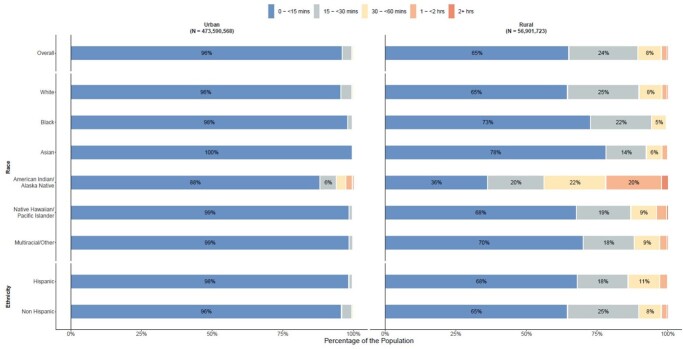
One-Way Travel Time to the Nearest 5y-11y COVID-19 Vaccination Site by Race, Ethnicity, Rurality

**Conclusion:**

We identified meaningful spatial patterns in the accessibility and uptake of pediatric COVID-19 vaccinations, including decreased vaccine uptake in areas of high SES and HCD vulnerability and greater uptake in areas of high MSL vulnerability. Our modeling and surveillance approaches are generalizable and can be applied to other emerging pathogen response and scarce resource allocation efforts.

**Disclosures:**

**Dena M. Bravata, MD, MS**, Castlight Health: Advisor/Consultant **Kathleen A. McManus, MD, MSCR**, Gilead Sciences, Inc.: Stocks/Bonds

